# Haematopoietic development and immunological function in the absence of cathepsin D

**DOI:** 10.1186/1471-2172-8-22

**Published:** 2007-09-26

**Authors:** Calogero Tulone, Yasuo Uchiyama, Marco Novelli, Nicholas Grosvenor, Paul Saftig, Benjamin M Chain

**Affiliations:** 1 Division of Infection and Immunity, UCL, London, UK; 2 Osaka University Graduate Medical School, Osaka, Japan; 3 Department of Histopatholology, UCL, London, UK; 4 Unit of Molecular Cell Biology and Transgenic Research, Institute of Biochemistry, Christian Albrecht University Kiel, Germany

## Abstract

**Background:**

Cathepsin D is a well-characterized aspartic protease expressed ubiquitously in lysosomes. Cathepsin D deficiency is associated with a spectrum of pathologies leading ultimately to death. Cathepsin D is expressed at high levels in many cells of the immune system, but its role in immune function is not well understood. This study examines the reconstitution and function of the immune system in the absence of cathepsin D, using bone marrow radiation chimaeras in which all haematopoietic cells are derived from cathepsin D deficient mice.

**Results:**

Cathepsin D deficient bone marrow cells fully reconstitute the major cellular components of both the adaptive and innate immune systems. Spleen cells from cathepsin D deficient chimaeric mice contained an increased number of autofluorescent granules characteristic of lipofuscin positive lysosomal storage diseases. Biochemical and ultrastructural changes in cathepsin D deficient spleen are consistent with increased autolysosomal activity. Chimaeric mice were immunised with either soluble (dinitrophenylated bovine gamma globulin) or particulate (sheep red blood cells) antigens. Both antigens induced equivalent immune responses in wild type or cathepsin D deficient chimaeras.

**Conclusion:**

All the parameters of haematopoietic reconstitution and adaptive immunity which were measured in this study were found to be normal in the absence of cathepsin D, even though cathepsin D deficiency leads to dysregulation of lysosomal function.

## Background

Cathepsin D, the first cathepsin to be identified, is an aspartic proteinase which is ubiquitously expressed in lysosomes of most eukaryotic cells. The enzyme's highly conserved sequence, and the fact that it is found in all eukaryotic organisms examined, suggests it plays an important role in cellular physiology, but its precise function is still poorly defined. The enzyme has a broad substrate specificity at acidic pH and was long thought to function in bulk degradative proteolysis of endocytosed or phagocytosed material. Raised cathepsin D levels are associated with metastatic potential in some cancers [1], perhaps reflecting the increased metabolism of these cells, or perhaps due to a role in extracellular matrix degradation. Cathepsin D has also been implicated in cell growth and apoptosis, though it remains unclear whether these functions require the proteolytic activity of the enzyme, or involve some separate ligand/receptor interaction [2].

In order to probe the role of cathepsin D further, a cathepsin D deficient mouse was previously generated by homologous recombination [3]. Cathepsin D deficiency conferred a lethal phenotype, with mice dying around days 21–22. The mice suffered severe weight loss, as well as neurological abnormalities including seizures and blindness. Histopathological investigation showed extensive atrophy of the digestive system, which is probably responsible for weight loss and death. The mice also showed profound atrophy of lymphoid tissue such as thymus and spleen. The precise link between cathepsin D deficiency and pathology remains unclear, but bulk proteolysis by cathepsin D deficient fibroblasts was found to be normal.

More recently, attention has shifted to the role of cathepsin D in neuronal tissues. As indicated above, cathepsin D deficient mice exhibit various neurological abnormalities, and these have been related to neuronal degeneration [4,5] associated with the development of lipofuscin containing granules, characteristic of a class of lysosomal storage diseases. The link between cathepsin D deficiency, lipofuscin containing granules (which may represent abnormal autophagic vacuoles) and neurological dysfunction has now been observed in several other species including Drosophila [6], sheep [7], dogs [8] and man [9,10]. The specific target of cathepsin D which is responsible for these pathologies remains unidentified, but the action of cathepsin D could be indirect via activation of a proenzyme. For example, cathepsin D has been proposed to play a role in epidermal differentiation via regulation of transglutaminase 1 activation [11].

There have been numerous suggestions that cathepsin D plays a role in cells of the immune system. Early studies proposed a role in antigen processing for the class II MHC endocytic pathway [12]. However, many of these studies have used the microbial proteinase inhibitor pepstatin to block cathepsin D. Interpretation of these experiments is difficult because pepstatin also blocks cathepsin E, a second non-lysosomal aspartic proteinase found in many cells of the immune system. More definitive *in vitro *studies using cathepsin D deficient mouse spleen cells suggested that this enzyme was dispensable for antigen processing/presentation [13]. More recent studies which combined pharmacological inhibition with the use of the cathepsin D deficient mice suggest that cathepsin E, rather than cathepsin D, is the major aspartic proteinase active in the antigen processing pathway [14]. Cathepsin D may also play a role in limiting antigen processing by destruction of antigen epitopes [15].

The function of cathepsin D in the development and function of the immune system remains unclear, since the multiple abnormalities and ultimate lethality seen in the enzyme deficient mice both limit the extent of immunological investigations, and complicate the interpretation of in vivo phenotypes. In this study, mice which selectively lack cathepsin D in cells of haematopoietic origin are generated by making bone marrow radiation chimaeras. This model is used to test the hypothesis that cathepsin D plays a non-redundant role in lysosomal function in immune cells, and examines the importance of this during development and function of the major branches of the adaptive immune system in vivo.

## Results

**Figure 1:**
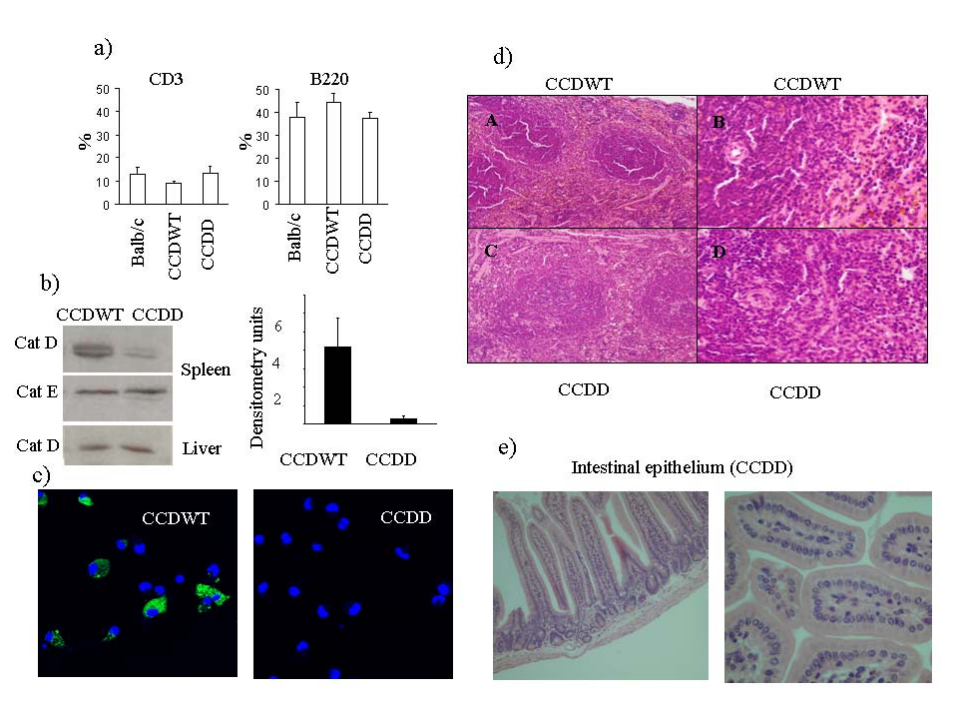
**Expression of cathepsin D and tissue histology in cathepsin D chimaeric mice**. a) Leukocytes cells were collected from control unirradiated mice (Balb/c), or from CCDWT or CCDD between 8 and 12 weeks post reconstitution, and stained for CD3 (T cells) or B220 (B cells). The results show the average % of each cell type, from a minimum of 5 different experiments, with standard error of mean. No significant differences (Student's T Test) were observed between any of the three groups of mice. b) Spleens and livers were collected from CCDWT and CCDD mice three-four months post reconstitution, and analysed for cathepsin D and cathepsin E expression by Western blot. The blot for spleen shows the presence of precursor (53KD) and single chain (48KD) forms of cathepsin D. The two single chain degradation products of 31 and 14KD are not shown in this figure. Left panel shows a representative example from one individual mouse from each group. Right panel shows the mean band density (and standard error of the mean) for cathepsin D staining for spleen extracts from five mice taken from three independent experiments. c) Dendritic cells were cultured from bone marrow of CCDWT and CCDD mice, and stained for cathepsin D expression by immunofluorescence. Nuclei are stained with DAPI. At least 100 cells per slide were scored as cathepsin D positive or negative from each sample. The % cells showing cathepsin D staining was greater than 80% for CCDWT, and less than 1% for CCDD mice. d) Spleen from CCDD and CCDWT mice was collected and standard haematoxylin and eosin staining was performed on 4 μm sections of formalin-fixed paraffin embedded tissues. A. Low power view of CCDWT mouse spleen. B. High power view of CCDWT spleen showing PALS (periarteriolar lymphoid sheath) and adjacent red pulp. C. Low power view of CCDD mouse spleen. D. High power view of CCDD mouse spleen showing PALS and adjacent red pulp. e) Upper intestine from CCDD mice was collected and processed as for panel d. Longitudinal and transverse sections show the normal crypt morphology of the adsorptive epithelium.

Irradiated mice which received bone marrow from either wild type or cathepsin D deficient mice all survived, while control irradiated mice which did not receive bone marrow all died. Full reconstitution as measured by blood T cell and B cell counts took two-three months, and blood leukocytes of chimaeras were routinely phenotyped by flow cytometry before use in experiments. No significant differences in numbers of T cells or B cells (fig 1a) or granulocytes (not shown) were observed between unirradiated control mice, or chimaeras generated from bone marrow taken from cathepsin D deficient (CCDD) or cathepsin D wild type (CCDWT) mice. The levels of cathepsin D in tissues collected from chimaeras were measured by Western blot (fig 1b). The antibody detects both the proenzyme (53KD) and mature single chain (48kD) enzyme (note two bands in fig 1b). Levels of cathepsin D (both proenzyme and mature) in spleen of CCDD chimaeras were less than 10% of those found in spleens of CCDWT chimaeras, confirming that most or all the spleen haematopoietic cells were derived from donor precursors, and did not represent the selective outgrowth of a small minority of surviving host precursor cells. In order to confirm that equal amounts of protein were present in both samples, the membranes were reprobed for cathepsin E, which was present in equal amounts in CCDD and CDDWT spleens (fig 1b). In contrast, levels of cathepsin D in liver, which is composed predominantly of non-haematopoietic tissue, were equivalent in both sets of chimaeras (fig 1b, bottom panel). Western blot of liver showed a single band corresponding to mature cathepsin D, suggesting that, in contrast to spleen, little proenzyme was stored in cells of this tissue.

Since spleen contains non-haematopoietic cells which may contribute to the remaining signal seen in Fig 1b dendritic cells were cultured from bone marrow of chimaeras and stained for cathepsin D expression by immunofluorescence (fig 1c). Over 80% of the dendritic cells from CCDWT chimaeras showed strong positive lysosomal staining with cathepsin D antibody, while in contrast, less than 0.1% of CCDD cells showed any staining. Spleen morphology in CCDWT and CCDD chimaeras was indistinguishable (fig 1d) (although chimaeric spleens were always smaller than unirradiated age matched control spleens), and there was no evidence of the extensive atrophy seen in the cathepsin D deficient donor mice immediately prior to death [3]. Intestinal morphology was also normal (fig 1e), and no atrophy was observed in chimaeras. These results suggest that the defect in intestinal development observed in cathepsin D deficient mice is an autonomous defect in intestinal cell function, rather than a consequence of haematopoietic dysfunction. In contrast, lymphoid tissue atrophy observed in cathepsin D deficient mice is a secondary effect, perhaps resulting from gut dysfunction.

**Figure 2:**
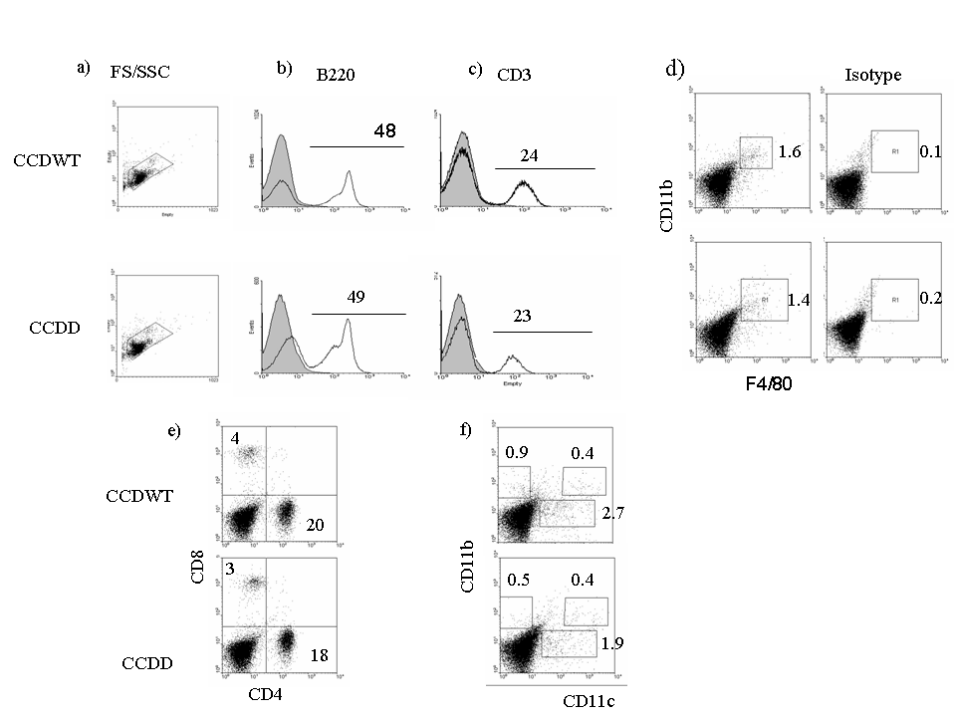
**Flow cytometry of major spleen leukocyte populations in CCDWT and CCDD**. Spleen cells were collected from chimaeras at least three months post-reconstitution, and stained for various lineage markers as described in Materials and Methods. One representative staining of at least 6 separate experiments on different mice of each chimaera type is shown. The % of cells staining for each type is shown above the marker (histograms), in each quadrant (e) or in the gate shown (d, f). An example of isotype control staining is shown in right column of panel d. a) Forward/side scatter plot for spleen cells showing normal distribution of size/granularity in both CCDWT and CCDD. Gate shows region analysed for plots b-f b) Single colour staining for B cells with anti-B220 antibody. c) Single colour staining for T cells with anti-CD3 antibody. d) Two colour staining for macrophages using anti-F4/80 and antibody anti-CD11b. e) Two colour staining with anti-CD4 and anti-CD8 antibodies. f) Two colour staining for dendritic cells with anti-CD11b and anti-CD11c antibodies.

**Figure 3:**
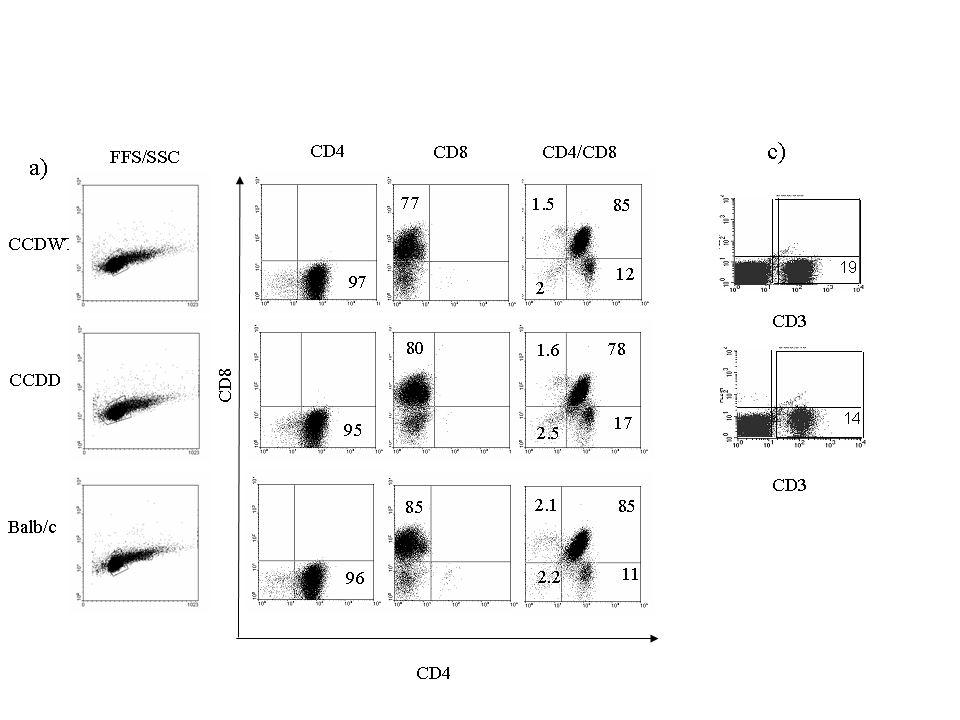
**Flow cytometry of major T cell populations in CCDWT and CCDD thymus**. Thymocytes were collected from chimaeras at least three months post-reconstitution, or a control age matched Balb/c mouse and stained for various lineage markers as described in Materials and Methods. a) Forward/side scatter plot for thymus cells showing normal distribution of size/granularity in both CCDWT and CCDD. Gate shows region analysed for plots b. b) Single and double staining with anti-CD4 and anti-CD8 antibodies. c) Single colour staining for T cells with anti-CD3 antibody. One representative staining of at least 5 separate experiments on different mice of each chimaera type is shown. The % of cells staining for each type is shown in each quadrant.

The cellular composition of lymphoid tissue and thymus was examined in more detail, as shown in figs 2 and 3. Spleen (and lymph nodes, not shown) of CCDWT and CCDD mice contained normal proportions of B cells (detected with B220 antibody), T cells (detected with CD3 antibody) and macrophages (double positive for CD11b and F4/80) (figs 2a–d). The proportion of CD4 versus CD8 cells, and the proportions of the various subpopulations of myeloid dendritic cells (CD11c^+^, CD11b^+ ^and CD11b^+^) were also the same in CCDWT and CCDD (figs 2e, f). The proportion of granulocytes in blood and spleen was also the same between experimental groups (not shown). Finally, the proportion of CD3, CD4, CD8 and double positive T cells in thymus was the same in Balb/c recipients, CCDWT and CCDD animals (figs 3a–c), suggesting T cell development was not affected by cathepsin D deficiency. Thus, cathepsin D is dispensable for the normal development and differentiation of all the major cell types of the immune system.

**Figure 4:**
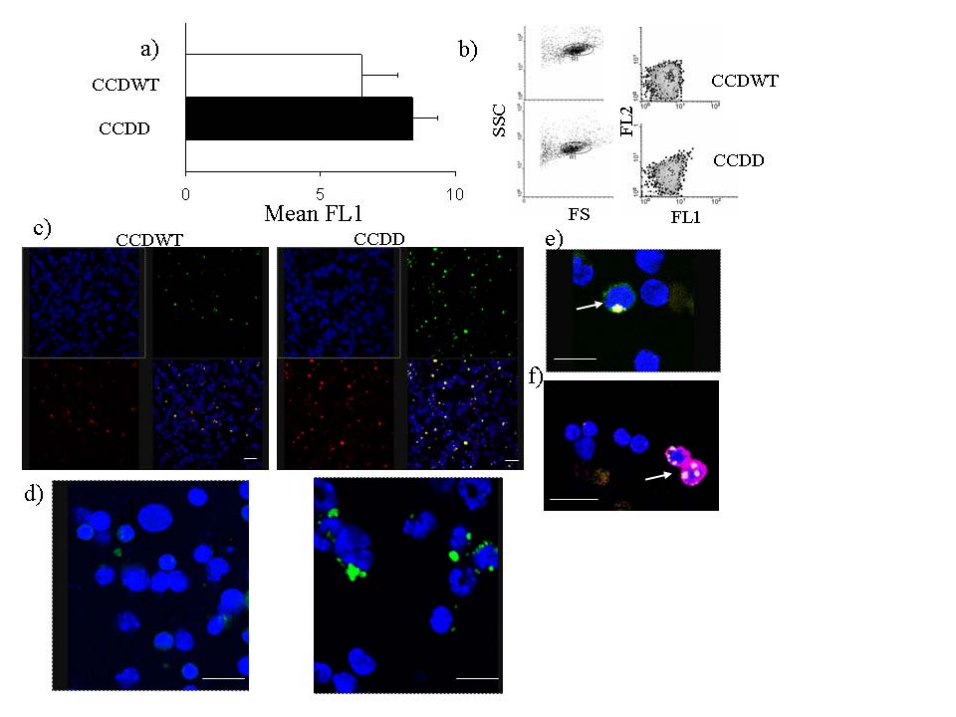
**Increase in autolysosomal (autophagic) vacuoles in CCDD spleen cells**. a) and b) Flow cytometry of unstained spleen cells from CCDWT and CCDD chimaeras. a) Average FL1 signal from five unstained preparation of spleen cells for CCDWT and CCDD. The average of CCDD is significantly greater than the CCDWT (Student's t-test two tailed, p < 0.02). b) Left panels show forward and side scatter profiles, which are indistinguishable between the two experimental groups. Right panels show contour plots of FL1 and FL2 autofluorescence. Note the different shape of the CCDD plot, reflecting increased fluorescence in both FL1 and FL2 channels. c) Spleen cells from CCDWT (left) and CCDD (right) were adhered to poly L-lysine, and fixed as described. Images were collected by confocal laser microscopy. The top left panel shows nuclear staining with DAPI, acquired using the UV laser. The top right panel shows images acquired using the Argon laser (excitation 488) and the emission settings for FITC fluorescence. The bottom left panel shows images acquired using the Argon laser and the emission settings for PE fluorescence. The bottom right shows a merged image of the three other panels. Scale bar – 10 μM. d) High power view of cells from CCDWT and CCDD. Scale bar – 10 μM. e) As for d, but arrow shows T cell stained with anti-CD3 FITC, containing autofluorescent granule. f) As for d, but arrow shows macrophage stained with anti-CD11b APC, containing autofluorescent granules.

**Figure 5:**
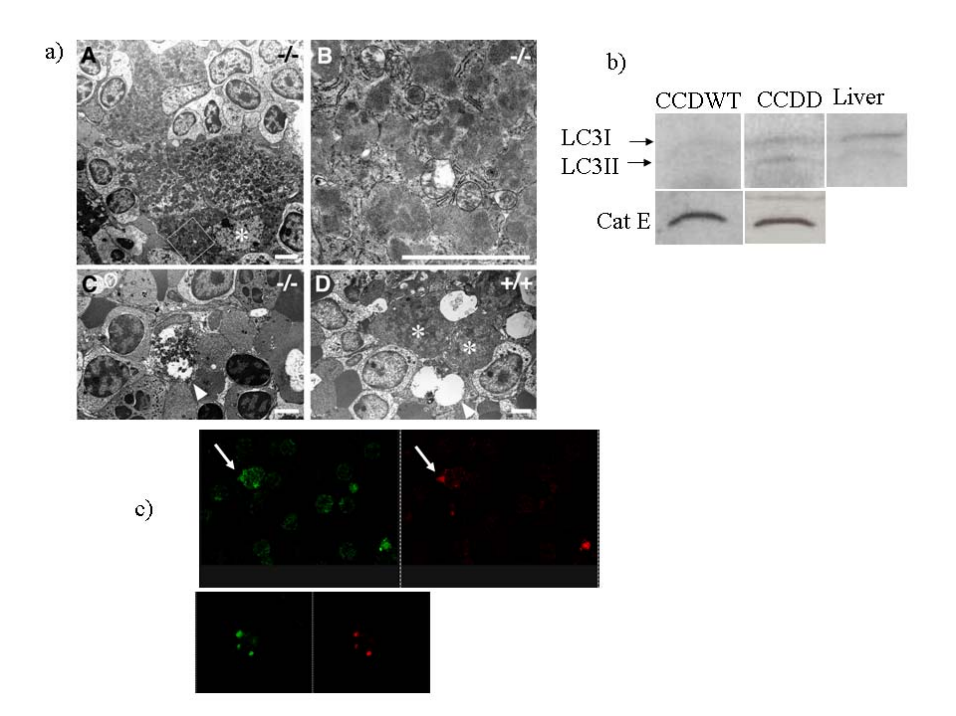
a) Electron microscope images of spleen cells from CCDD (A-C) and CCDWT (D). Macrophages (one example marked with asterisk in panel A) in the marginal region of the white pulp contain abundant lysosomal/vacuolar structures, most of which resemble granular osmiophilic deposits (B, a higher power of the quadrated area in A). Lymphocytes in the region appear intact (A). In the red pulp, such macrophages with abundant lysosomal structures (arrow) appear to be less in number (C). Macrophages (asterisk) and lymphocytes in the spleen of CCDWT show normal structures (D). b) Western blot showing expression of LC3 and cathepsin E (as loading control) in spleens from CCDWT and CCDD. A blot of CCDWT liver with LC3 antibody is shown for comparison. Arrows show the position of the soluble (LC3I – 18kD) and membrane bound (LC3II – 16kD) forms of LC3. c) Distribution of LC3 in spleen cells from CCDD (LC3 was not detectable above background in CCDWT). Left panel – LC3 staining. Right panel – autofluorescence. Note presence of high levels of LC3 in a cell with strong autofluorescent granules (arrow). Lower panels shows autofluorescent granules in controls, without primary antibody.

In the course of carrying out the flow cytometry studies shown in figs 2 and 3, we noted that spleen cells, lymph node cells and thymus cells from CCDD mice consistently showed a small but significant increase in autofluorescence in the absence of any antibody (typically increase of around 20% in background FL1, see fig 4a, p < 0.02, n = 5), which was observed in both FL1 and FL2 channels (fig 4b). Since increased autofluorescence is characteristic of the lipofuscin deposits previously noted in cathepsin D deficient neurones [16], we examined this phenomenon further. Cytospin preparations of spleen cells from chimaeric mice were examined by confocal microscopy (figs 4c–f). Cells from CCDD mice were found to contain strongly autofluorescent granules at a considerably higher frequency than those of CCWT. The granules were of various sizes, and cells typically contained one or two granules only (fig 4d). The granules were found both in CD3 T cells (Fig 4e) and CD11b positive macrophages (Fig 4f), but rarely in B cells (not shown). Spleen cells from the chimaeras were also examined by electron microscopy (fig 5a). Macrophages located in the marginal areas of the white pulp from CCDD chimaeras contained many large vacuoles with significant amounts of undigested substances. These structures resembled granular osmiophilic deposits, typical of neuronal ceroid-lipofuscinosis autophagic or autolyososomal bodies [17]. A biochemical marker of autophagy is microtubule-associated protein 1 light chain 3 (LC3)[18]. Spleen cells from CCDD chimaeras showed increased levels of LC3 protein, as detected by Western blot analysis (fig 5b). In contrast to the results obtained in liver and brain [19], LC3 protein expression in CCDWT was very low, and cathepsin D deficiency was associated with increased expression of both light (LC3II, membrane-bound) and heavy (LC3I, cytosolic) forms of LC3. The proportion of membrane form was higher in the CCDD spleen than that seen in control liver tissue (fig 5b, left panel). As expected, no differences in LC3 expression were observed between the livers of CCDD and CCDWT (not shown). LC3 distribution was also investigated by immunofluorescence. The LC3 antibody stained discrete intracellular vesicles, consistent with an autophagosome distribution (fig 5c, left panel). LC3 staining was often high in cells with autofluorescent granules (Fig 5c, right panel), although since autofluorescence was visible in both red and green channels, it was not possible to definitively determine that LC3 was present in the autofluorescent granules themselves.

**Figure 6:**
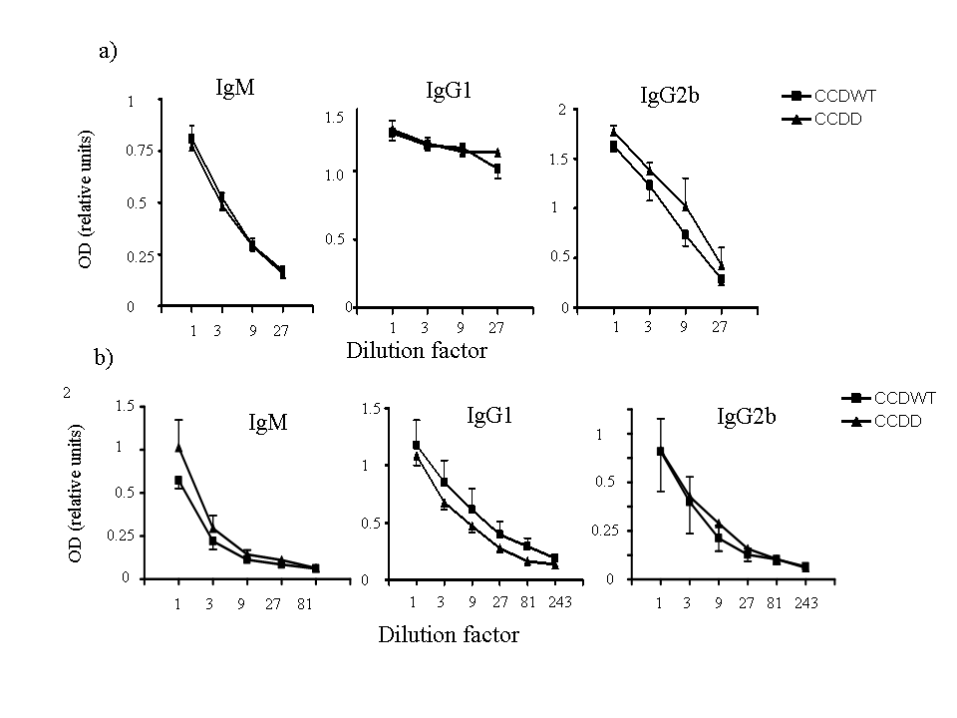
**Antibody responses to soluble and particulate antigens in CCDWT and CCDD**. a) ELISA from chimaeric mice immunised with 6 μg DNP-BIg/alum as described in the text. IgM responses were measured five days post priming; the highest concentration of plasma corresponds to a dilution of 1: 30. IgG1 and IgG 2b responses were measured six days post boost; the highest concentration of plasma corresponds to a dilution of 1: 100. Each point represents average and standard error of the mean of three mice. Preimmune sera all gave OD values of less than 0.2 at the highest concentration shown. b) ELISA from plasma of chimaeric mice immunised with SRBC (10^8 ^cells/mouse). IgM responses were measured five days post priming; the highest concentration of plasma corresponds to a dilution of 1: 300. IgG1 and IgG 2b responses were measured six days post boost; the highest concentration of plasma corresponds to a dilution of 1: 900. Each point represents average of at least six mice. Preimmune sera all gave OD values of less than 0.1 at the highest concentration shown.

The functional integrity of the immune system from cathepsin D deficient mice was then tested *in vivo*, using two different antigen systems. The first was the antibody response to DNP-Bovine gamma globulin (DNP-BIg), a classical soluble T helper dependent antigen, administered with alum adjuvant. Mice were immunised with a range of concentrations (0.1–10 μg/ml) and antibody response (IgM, and various IgG subclasses) was measured after a primary and secondary challenge. Representative examples of the data showing IgM responses after primary immunisation, and IgG responses after secondary immunisation, are shown in fig 6 (immunization with 6 μg is shown, since this was the lowest concentration found to give reproducible responses for all isotypes tested). The response did vary between individual mice. However, both CCDWT and CCDD mice showed good responses as shown. A two-way ANOVA on the data from all the animals showed that cathepsin D deficiency did not significantly affect the antibody response for IgM and IgG1 at the concentrations tested, but had a slight enhancing effect on IgG2b (p = 0.03).

Since cathepsin D is a phagolysosomal enzyme, we reasoned that cathepsin D might play a more significant role in responses to particulate antigens. Chimaeric mice were therefore immunised with SRBC, and antibody responses measured as before (fig 6b). Once again, both CCDWT and CCDD chimaeras showed strong responses to the antigen. Two-way ANOVA showed no significant effect of cathepsin D deficiency on IgM, IgG1 or IgG2b (P > 0.05).

**Figure 7:**
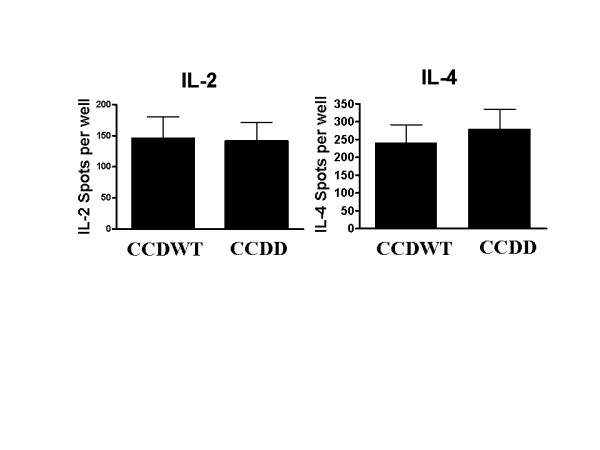
**T cell responses to particulate antigen in CCDWT and CCDD**. Spleen cells (1.2 × 10^6^) from chimaeric mice from each experimental group immunised with SRBC (10^8 ^cells/mouse) were cultured for eight hours in the presence of SRBC (4 × 10^5^) and the number of cells secreting IL-2 or IL-4 measured by ELISPOT. The results show mean and SEM number of spots from eight individual mice. Spleen cells from mice immunised with PBS only gave values of less than 10 spots/well for IL-2 and less than 80 spots per well for IL-4.

The T cell response to SRBC was assessed in parallel by measuring IL-2 and IL-4 release by spleen cells from immunised mice using ELISPOT (fig 7). No significant interferon γ release was observed in any mice, reflecting the known TH2 bias of the Balb/c strain. We observed no significant differences between CCDWT and CCDD mice for either IL-2 or IL-4 release (p > 0.2, Student's T-Test).

## Discussion

Cathepsin D, consistent with its highly conserved sequence and ubiquitous expression throughout the eukaryotes, is an essential non-redundant enzyme. As well as contributing to lysosomal proteolysis, the enzyme has been implicated in the apoptotic signalling pathway [20,21] and in regulation of cell proliferation [22]. Consistent with such wider roles, previous studies have reported extensive tissue pathology associated with cathepsin D deficiency. These have included abnormal gut development [3], atrophy of primary and secondary lymphoid tissues [3] and most notably extensive neurodegeneration and neuronal cell death [23]. Previous studies, however, have been limited to models of global, rather than tissue specific cathepsin D deficiency. The ubiquitous distribution of cathepsin D presents some difficulties in the interpretation of such models. In particular, cell damage may occur from a direct effect of absence of cathepsin D on that cell's function (intrinsic), or as a result of secondary damage due to malfunction of other cells or organs (extrinsic). This problem has been circumvented in part by studying the function of specific cell types in vitro [24,25], but this approach is limited in the mouse, because animals carrying the cathepsin D deficiency are non-viable after three weeks of age.

The use of bone marrow reconstitution to make chimaeras is an alternative approach to study the intrinsic effects of cathepsin D deficiency on haemopoetic cell development and function. In contrast to the results reported by Saftig et al. [3], the lymphoid tissues of these mice reconstitute normally, although we cannot rule out the possibility that there are undetected abnormalities in eosinophils, megakaryocytes or some other cell type we have not yet investigated. We conclude that the atrophy of lymphoid organs reported previously is therefore a secondary defect, arising perhaps from failure of the digestive system and gut atrophy. Cell recoveries of the major populations of lymphocytes and macrophages were also normal, suggesting that the absence of the enzyme did not affect cellular homeostasis. Consistent with these results, no major differences in number of apoptotic cells was observed in the cathepsin D deficient mice (data not shown). A recent study has suggested that cathepsin D may mediate macrophage apoptosis in response to bacterial challenge [26]. Studies are in progress to investigate this phenomenon in the chimaeric model.

Our studies have also suggested that the major branches of the adaptive immune system function normally in the absence of cathepsin D, although we cannot rule out that abnormalities exist in innate immunity, or other parameters of adaptive immunity not yet tested. These studies are the first to investigate immune function in the absence of cathepsin D in vivo. Previous studies on antigen processing have demonstrated small or no effects on antigen processing in vitro [27,28]. Further studies investigating the adaptive immune response to intracellular pathogens are in progress to explore whether cathepsin D may have a more selective processing or even a direct microbicidal role in control of such pathogens [29,30].

Numerous studies have reported a defect in autophagic function in cells lacking cathepsin D [31]. In agreement with these studies, we find an increased number of autophagic vacuoles in the spleen cells of deficient mice. However, in contrast to the neuronal models, this defect appears to be of limited extent. Only a small proportion of cells show the presence of autophagic vacuoles, and there is no evidence that the defect leads to extensive cell death. This may reflect a much greater sensitivity of neuronal cells to the build up of undigested additional enzyme redundancies in the cells of the immune system, leaving them more able to cope with the absence of cathepsin D [32,33]. However, there is considerable current interest in the role of autophagy in the antigen processing/presenting pathway for endogenous antigens [34], and the chimaeras described in this paper will provide a valuable model to see if cathepsin D has a specific role in this pathway.

## Conclusion

In this study, we describe a model in which the role of cathepsin D in cells of the immune system can be examined in vivo, in the absence of secondary damage due to malfunction of other tissues. These experiments clearly demonstrate that the major cellular components of the immune system can develop in the absence of endogenous cathepsin D, and that adaptive immunity to both soluble and particulate antigens is broadly intact. Further studies will be required to determine whether more subtle inhibitory or enhancing effects of cathepsin D on T cell dependent immune responses can be observed in vivo, mirroring the effects noted *in vitro *[35,36].

The lymphoid tissue of these cathepsin D deficient chimaeras show evidence of the same lysosomal dysfunction which has been shown to give rise to major malfunction in the nervous system. The precise molecular link between absence of cathepsin D and the development of autofluorescent autolysosomal vesicles is still unknown, but it is certainly possible that further, more detailed examination of immune function in these chimaeras may yet identify further specific defects in responses to particular pathogens. The model system described in this study will allow the study of such defects to be extended from the *in vitro *models used previously to *in vivo *studies in the context of a functioning immune system.

## Methods

### Mice

Balb/c mice (Balb/c Ola Hsd) were purchased from Harlan UK and kept in the Biological Services, UCL. Mice carrying a neomycin insertion in the cathepsin D gene [3] were bred onto the Balb/c background for at least ten generations, and then maintained as a heterozygote inbred colony. Homozygotes could be identified by day 19 or 20 by their small size, and reduced mobility. Genotype was confirmed in each case by PCR as described [3]. All experiments were carried out under UK Animal Project Licence authorization.

### Bone marrow radiation chimaeras

Recipient Balb/c mice were kept on acidified water (0.01% conc. HCl in H_2_0) for 1 week prior to transfer. The mice were irradiated with 8 Gy (delivered over 15–30 minutes) with an X-ray source (A.G.O. HS X-RAY SYSTEM, Reading, UK) and then allowed to recover for 4–5 hours, before receiving 2 × 10^6 ^bone marrow cells from cathepsin D deficient, or wild type litter mates, intravenously in 0.2 mls PBS. One control mouse received PBS in each experiment. All these controls died within ten days of irradiation. The chimaeric mice were maintained for a minimum of 2–3 months in order to allow full reconstitution of the immune system (see Results).

### Dendritic cell culture

Mouse myeloid dendritic cells were obtained by culture of bone marrow cells (5 × 10^5^/ml) in Iscove's medium (Gibco BRL (Invitrogen Life Technologies/Life Sciences), Paisley, UK), 10% FCS (Gibco BRL) with the addition of GM-CSF (20 ng/ml; PeproTech, Rocky Hill, NJ, USA). Fresh medium and cytokine were added on day 4 and dendritic cells were harvested on days 7/8. Dendritic cells were further purified by magnetic cell sorting, using mouse CD11c^+ ^microbeads (CD11c (N418), Miltenyi Biotec, Bergisch Gladbach, Germany) and the appropriate columns (MS separation columns, Miltenyi Biotec), according to the manufacture's guidelines. The enriched population was 85–95% CD11c^+^.

### Western blotting

Western blots were performed on liver or spleen homogenate, prepared using a Dounce homoginiser. 30 μg protein (measured by Bradford assay) were loaded in each lane and separated by 12% denaturing SDS polyacrylamide gel electrophoresis (PAGE) under reducing conditions. Proteins were transferred electrophoretically to nitrocellulose membranes and then immunostained using standard procedures. Primary antibodies used were: monoclonal rat anti-mouse Cathepsin D (R&D System, Minneapolis, MN, US, 4 μg/ml); rabbit anti-rat Cathepsin E antibody (WAKO, Neuss, Germany), diluted 1:1000; rabbit anti-rat microtubule-associated protein 1 light chain 3 (LC3) antibody, diluted 1:1000 [37]. Primary antibodies were detected using HRP-conjugated rabbit anti-rat IgG or swine anti-rabbit IgG (DAKO, Glostrup, Denmark, diluted 1:2000), and ECL detection reagent (Amersham Pharmacia Biotech, Bucks, UK). For quantification, gels were scanned and digital images analyzed using Image J software [38].

### Immunofluorescence

Dendritic cells (5 × 10^4^) or spleen cells prepared as described above were allowed to adhere for 30 minutes on poly L-lysine (Sigma Aldrich, Poole, UK, 100 μg/ml in water) coated 18 mm circular cover slips. The cells were fixed for 10 minutes in 4% paraformaldehyde, permeabilised with 0.2% Triton-X for three minutes, and then stained using standard protocols for indirect immunofluorescence. Cathepsin D was detected with goat anti-mouse Cathepsin D antibody (1:100, R&D), and rabbit anti-goat FITC (DAKO). For detection of autofluorescent granules (fig 5) spleen cells (10^5^) were adhered to cover slips as above and fixed with paraformaldehyde, with no permeabilisation or further staining steps. For staining of LC3 the spleen cells were fixed for 10 minutes in 4% paraformaldehyde, permeabilised with 0.2% Triton-X for three minutes, and then stained using standard protocols for indirect immunofluorescence as above. Nuclei were counterstained with 4'-6-Diamidino-2-phenylindole (DAPI, 2 μg/ml) for ten minutes prior to mounting.

### Histology and histochemistry

Standard haematoxylin and eosin staining was performed on 4 μm sections of formalin-fixed paraffin embedded tissues.

### Flow cytometry

Cells were collected from spleen, thymus or lymph node of bone marrow radiation chimaeras, and stained using standard flow cytometry protocols. Fc receptor and non-specific binding was blocked by incubation in PBS containing 10% v/v rat serum (Gibco BRL) and 0.1% NaN_3 _for 45 min at room temperature. Primary antibodies used were: control FITC IgG_2b_,κ, PharMingen, Temse, Belgium; control PE IgG_2b_,κ Pharmingen (other isotypes were also tested and gave identical profiles, not shown); B220-FITC (CD45R) IgG_2a_,κ, PharMingen; CD3εFITC, IgG_2b_,κ, PharMingen; CD4-PE IgG_2a_, Caltag Lab, Paisley, UK and/or CD4APC (GK1.5) IgG_2b_,κ, PharMingen; CD8α/Ly-2-PE, IgG_2a_,κ, eBioscience; CD11b-APC (M1/70) IgG_2b_,κ, Biolegend; CD11c-PE (N418) IgG, eBioscience, Middlesex, UK; GR1-FITC (Ly-6C/G), IgG_2b_,κ, PharMingen; F4/80-PE, IgG_2a_,κ, eBioscience. Optimum concentrations were determined by titration. After staining, all samples were fixed by adding 100 μl 3.7% v/v formaldehyde/saline and stored at 4°C in the dark for analysis within 5 days on a FACScan (Becton-Dickinson, Mountain View, CA, USA) using WinMDI (Joseph Trotter, Scripps Research Institute, La Jolla, CA, USA or Cell Quest) software. For each sample not less than 10000 events were acquired.

### Immunization of bone marrow radiation chimaeras

Preimmune plasma was collected by tail bleed of all mice one day before immunization. Blood was collected in heparin-coated tubes (Microvette^® ^CB 300, Sarsted, Germany), and plasma was separated by centrifugation for 10 minutes at 10000 g, and stored at -20°C (in some experiments serum was collected instead of plasma, but results were always similar). All preimmune samples were tested in parallel to test samples, but were all found to give responses similar to background (data not shown).

Mice were then immunised with dinitrophenylated bovine Ig (DNP-BIg, [39]) or sheep red blood cells (SRBC). Chimaeric mice or unirradiated Balb/c controls were injected intraperitoneally with varying amounts of DNP-IgG (see below) in 100 μl PBS mixed with 100 μl adjuvant (Imject^® ^Alum, Pierce, USA), or with 500 μl of 2 × 10^8 ^cell/ml SRBC suspension (TCS Biosciences, UK). Plasma samples were collected 5/6 days after priming (primary response), and stored as above. 2 weeks after priming, the mice were boosted with the same amount of antigen as used during the first injection. 6 days after the boosting (secondary response), plasma was collected and stored as above. Mice immunised with SRBC were then sacrificed, and spleens collected for ELISPOT analysis as described below. Controls were injected with PBS alone.

### ELISA

DNP-IgG: 96 well ELISA plates (Nunc, Life Technologies, UK) were coated overnight at 4°C with DNP-BIg (10 μg/ml) in hydrogen carbonate buffer (0.1 M Na HCO_3_, pH 8.5). SRBC: 96 well ELISA plates were coated with 10 μg/ml poly-L-lysine (Sigma-Aldrich) in PBS for 30 minutes at room temperature. The plates were then washed with PBS and 50 μl freshly washed SRBC (2 × 10^6 ^cells/ml in PBS) were added. The plates were centrifuged at 600 × g for 10 minutes and 50 μl 0.5% (v/v) glutaraldehyde (electron microscopy grade, Sigma-Aldrich) in cold PBS were added and left for 20 minutes at room temperature. After 3 washings with PBS, wells were blocked with 200 μl of 1% (w/v) milk powder in 100 mM glycine (Sigma-Aldrich) for 30 minutes at room temperature. After 3 further washings in PBS, 300 μl blocking buffer was added to the wells and the plates were stored at -20°C until use.

### ELISA detection protocols (both antigens)

The plates were thawed, washed twice with 200 μl of 0.5% biotin free BSA (Sigma-Aldrich)-PBS and blocked with 200 μl of 0.5% biotin free BSA-PBS for 1 h at 37°C. Preimmune or test plasma was added at various dilutions, and incubated for 2 h at 37°C. The plates were washed 4 times with PBS. IgM and IgG subclasses were detected using mouse immunoglobulin isotype specific monoclonal antibodies : anti-IgM IgG_2a_,κ, PharMingen; anti-IgG2a, IgG_1_,κ, PharMingen; anti-IgG2b, IgG_2a_,κ, PharMingen; anti-IgG1, IgG_1_,κ, PharMingen; Binding was detected using alkaline phosphatase conjugated streptavidin (Caltag Lab), diluted 1:2000 in 0.5% biotin free BSA-PBS, and p-nitrophenyl phosphate (Sigma-Aldrich) prepared according to the manufacture's guidelines. The plates were developed at 37°C and absorbance read at 405 nm (ELISA plate reader, Revelation software) after 15–20 minutes.

### ELISPOT

Spleen cells (1.2 × 10^6 ^per well) from mice immunised with SRBC or unimmunised controls were cultured with washed SRBC (4 × 10^5^)in RPMI medium containing 5% FCS for eight hours in ELISPOT plates precoated with anti-IL-2 or anti-IL-4 antibody (eBiosciences, 5 μg/ml). Spleen cells were lysed by washing in water, and then the spots were developed using biotinylated anti-cytokine antibodies (eBiosciences, 1 μg/ml) and streptavidin alkaline phosphatase substrate as described in detail elsewhere [40].

### Electron microscopy of liver and spleen

Parts of liver and spleen samples obtained from chimaeric and control mice were cut into small pieces and fixed in 2% paraformaldehyde-2% glutaraldehyde buffered with 0.1 M phosphate buffer for 24 hours. The samples were postfixed with 2% OsO4, dehydrated with a graded series of alcohol, and embedded in Epon 812. Ultrathin sections were cut with an ultramicrotome (Ultracut N, Hitachi, Japan), stained with uranyl acetate and lead citrate, and observed with a Hitachi H7100 microscope.

## Abbreviations

CCDD, chimaeric cathepsin D deficient; CCDWT, Chimaeric cathepsin D wild type

## Competing interests

The author(s) declares that there are no competing interests.

## Authors' contributions

CT is responsible for experimental work producing for all data except as detailed below. fig 7. YU is responsible for the electron microscopy shown in fig 5a, and for production of antiserum to LC3. MN is responsible for figs 1d and 1e. PS is responsible for provision of cathepsin D deficient mouse. NG is responsible for figs 4e, f, 5c. BC is responsible for overall project management, funding, and drafting of manuscript, and for providing data for figs 4c, fig 7. All authors read and approved the final manuscript.
